# Predictors of students’ academic achievements in allied health professions at King Saud University: a retrospective cohort study

**DOI:** 10.1186/s12909-021-02525-x

**Published:** 2021-02-06

**Authors:** Sultana A. Alhurishi, Ghadeer S. Aljuraiban, Fahdah A. Alshaikh, Mona M. Almutairi, Khalid M. Almutairi

**Affiliations:** 1 Department of Community Health Sciences, College of Applied Medical Sciences, King Saud University, Riyadh, Saudi Arabia; 2 Department of Statistics and information, Vice Rectorate for Planning and Development, King Saud University, Riyadh, Saudi Arabia

**Keywords:** Medical education, Allied health professions, Admission criteria, Saudi Arabia, Aptitude tests, Achievement tests

## Abstract

**Background:**

The admissions criteria for colleges of medicine and allied health professions include several cognitive predictors. Little is known of the admissions criteria for the allied health professions and their correlation with students’ academic performance. This study investigates predictors for students’ academic achievements at allied health colleges at King Saud University.

**Design:**

Retrospective cohort study.

**Settings:**

College of Applied Medical Sciences, College of Nursing, and Prince Sultan bin Abdulaziz College for Emergency Medical Services, Saudi Arabia.

**Participants:**

The sample comprised 1634 students.

**Method:**

The high school grade average (HSGA), aptitude test (APT) score, achievement test (ACT) score, and current grade point average (GPA) were retrieved. The data were analysed using Pearson’s correlation coefficient and regression analysis.

**Results:**

HSGA, ACT, and APT were significantly positively associated with students’ academic performance in colleges for all allied health professions. Multivariate regression analysis showed that the most predictive variable for all allied healthcare professions was HSGA (β = 0.347), followed by ACT (β = 0.270) and APT (β = 0.053) scores. The regression model indicated that the HSGA, APT, and ACT together predicted 26.5% of the variation in students’ cumulative GPAs at the time of graduation.

**Conclusion:**

The admissions criteria for the allied health colleges at King Saud University predicted only 26.5% of the students’ cumulative GPA at the time of graduation. Other noncognitive admission criteria should be taken into consideration to improve the prediction of students’ academic potential.

## Background

For decades, the admissions process for colleges of medicine and allied health professions has been a topic of interest in the area of higher education. Universities have invested large amounts of money in developing admission criteria with the aim of selecting appropriate candidates and maintaining a high educational standard [1]. The practice of applicant selection varies greatly among institutions and because of the increased number of applicants, it is more detailed now than ever before. Undergraduate education in allied health professions plays an influential role in guiding students to reach their future roles with high clinical skills and to use them to support the health and well-being of those in their community who are in need for health care. The selection criteria are designed to find individuals with sufficient intellectual abilities and personalities to meet the challenges encountered in obtaining a degree in an allied health profession [2]. Selection methods vary between qualitative methods such as interviews, personal statements, or emotional intelligence. The more common methods include quantitative approaches using preadmission grades [3]. Selection methods are often based on previous academic performance, aptitude tests, interviews, and personal statements [2].

The use of these methods varies. Globally, the selection of undergraduate students involves two main phases [4]. The goal of the first phase is to reduce the number of applicants. For this purpose, aptitude tests, academic records, personal statements, references, situational judgment tests, personal assessments, and emotional intelligence tests are used. During the second phase, the suitability of the candidate to study at a health-related college is assessed through a range of approaches or a combination of them—including traditional interviews, structured interviews, multiple mini-interviews, and selection centers—using work samples [4]. Fair and robust selection criteria are therefore required to select suitable candidates [5].

In Saudi Arabia, students complete 12 years of formal education before application to university admission. Admission criteria for colleges of medicine and allied health professions include cognitive criteria, assessed using the following weighting: 30 % for cumulative high school grade point average at application (HSGA), 30 % for aptitude test (APT) score, and 40 % for achievement test (ACT) score [6]. The HSGA is a cumulative score that is calculated at the high-school level. The National Centre for Assessment of the Ministry of Higher Education introduced the Saudi National Aptitude and Achievement Exams in 2002 [7]. The APT is a standardized test that assesses analytical and deductive skills in verbal and quantitative sections, and the ACT is a standardized test that assesses comprehension, application, and inference in biology, chemistry, physics, English, and mathematics. King Saud University is one of the oldest public universities in Saudi Arabia, receives a large number of applications each year, and they are sorted according to their weighting. The cut-off value of the weighting varies each year in relation to the number of candidates and the capacity of the university. Applicants who surpass the minimum weighting value are evaluated for noncognitive skills and must pass a face-to-face interview. Although the interview facilitates the evaluation of the attitudinal and motivational characteristics of the candidates, it is not weighted in the overall process.

An important question arises in relation to whether the admissions criteria can predict students’ academic performance. Edwards et al. (2013) argue that while admissions criteria help institutions select students, they do not aid in predicting their performance once admitted [2]. Several studies have examined the methods used as admissions criteria in international medical colleges and in Saudi Arabia [4]. The admissions criteria for medical colleges have also been assessed in Saudi Arabia [6–12]. Alhadlaq et al. (2015) studied the suitability of admission criteria to predict students’ academic performance at colleges of medicine, dentistry, and pharmacy in their early years of study at King Saud University [6]. They found that ACT and HSGA were effective predictors of students’ early academic performance, but APT was not a strong predictor. However, it is not known if this prediction would appear in colleges of allied health professions programs. Furthermore, several factors play fundamental role on academic achievements of allied healthcare professionals’ students. For example, students’ profile such as age, gender and language, students’ affective factors, academic related factors, environmental factors, academic outcomes and psychological outcomes are found predictors associated with academic success among nursing students [13]. The complexity and multifactor interaction between the students and academic institution led to experience different academic outcomes [3]. Although, the association between these factors and the selection of suitable candidates-based on entry requirements predetermined criteria need to deeply understood.

These programs are similar in length because all of them feature one preparatory year, followed by three years of study and a one-year internship. The unified system of cumulative calculation of Grade Point Average (GPA) on a scale of 1 to 5 facilitated comparisons across allied health professions programs. It has been questioned whether the admissions criteria for allied health professions programs predicts students’ academic performance, as measured by their cumulative GPA at the time of graduation, specifically in Saudi Arabia. Thus, we hypothesized that preadmissions criteria (using HSGA, ACT and APT) would predict the success of students at the time of graduation. This study investigated the association between admissions criteria at King Saud University and cumulative GPA at the time of graduation for three colleges of allied health professions, namely, the College of Applied Medical Sciences (CAMS), the Nursing College, and the Prince Sultan bin Abdulaziz College for Emergency Medical Services (PSCEMS) in Saudi Arabia.

## Methods

### Study design

A retrospective cohort observational study was conducted at King Saud University. The university offers 13 undergraduate programs related to allied health care professions through three main colleges. The Nursing College offers a nursing program; PSCEMS offers an emergency medical services program; and CAMS provides a broad range of 11 programs, namely, physiotherapy, optometry, radiological sciences, biomedical instrumentation, health education, clinical nutrition, dental technology, dental hygiene, clinical laboratory sciences, speech therapy, and occupational therapy. All of these programs required passing four years of study following by a one-year internship. Ethical approval was obtained from Ethics Committee Review Board at King Saud University (ref: 19/0747/IRB).

### Data collection

In November 2019, the study data were retrieved from King Saud University with the assistance of the Statistics and Information Department, which has access to the records of all graduate students. The data pertained to three graduate college cohorts from CAMS, the Nursing College, or PSCEMS who graduated between the 2016–2019 academic years. The sample comprised a total of 1979 graduates. It is worth noting that the number of graduates increased each year: 533 in 2016–2017, 687 in 2017–2018, and 759 in 2018–2019.

### Variables

Data retrieved include the students’ gender, college name and program of study, GPA, HSGA, APT, ACT and year of graduation. The academic achievement was defined using the cumulative GPA at the time of graduation. It was calculated using the mean grade at the point of graduation. The main independent variables were HSGA, APT, and ACT. Other independent variables were gender, program and college.

The weighting ratio was calculated using the following formula: (Weighting ratio = (HSGA × 0.30) + (APT × 0.30) + (ACT × 0.40))

### Statistical analysis

Statistical analysis was done out using the Statistical Package for Social Sciences (SPSS) version 23 (SPSS Inc., Chicago, IL, USA). Data were processed using a descriptive analysis of frequencies, percentages, and means and standard deviations. The Levene’s test of equality of variances was used as part of t-test and analysis of variance to examined the homogeneity of variance. Analytical processes including univariate and multivariate linear regression were used to predict academic achievement between the GPA and independent variables such as HSGA, APT, and ACT gender, program and college for the study cohort. A *p* – value of < 0.05 was considered statistically significant for all analyses. Following this, the relationship between GPA and independent variables was assessed using multivariate linear regression for the study cohort and each program.

## Results

### Characteristics of the study sample

A total of 1979 records were available, however, screening found 345 incomplete records. To limit the uncertainty arising during data analysis because of missing values, records with missing information were excluded from the analysis. One thousand six hundred thirty-four complete graduate records were enrolled in this study. More than half of the graduates (51.1 %) were male and majority of the graduates studied at CAMS (75.9 %), then College of Nursing (14.4 %) and PSCEMS (9.7 %), as shown in Table 1. Nursing program graduates were the highest percentage (14.4 %), followed by clinical nutrition (12.7 %), emergency medical services (9.7 %), health education (9.7 %), clinical laboratory sciences (9.6 %) and radiological sciences (9.1 %). The number of graduates has increased over the years, from 371 graduates (2016–2017) to 731 in (2018–2019). The Levene’s test for equality of variances was not significant; the absolute value of t indicates the groups were statistically not different.

**Table 1:** Description of the study cohort (n = 1632)

Variable	Frequency	Percentage
**Sex**		
Male	835	51.1
Female	799	48.9
**College**		
CAMS	1240	75.9
Nursing	235	14.4
PSCEMS	157	9.7
**Undergraduate Program**		
1. Nursing	235	14.4
2. Emergency Medical Services	157	9.7
3. Health Education	158	9.7
4. Clinical Nutrition	207	12.7
5. Clinical laboratory sciences	157	9.6
6. Radiological sciences	149	9.1
7. Physiotherapy	140	8.6
8. Optometry Doctor	84	5.1
9. Dental Hygiene	80	4.9
10. Dental technology	50	3.1
11. Occupational Therapy	71	4.3
12. Biomedical Instrumentation	52	3.2
13. Speech Therapy	92	5.6
**Year of Graduation**		
2016-17	371	22.7
2017-18	530	32.5
2018-19	731	44.8
Total	1632	100

The means and standard deviations for the cumulative GPA of the study cohort at graduation was 4.17 ± 0.48, out of a scale score range of 2.47–5.0. The mean scores for HSGA, APT, and ACT for the cohort were 97 ± 2.78, 81.3 ± 5.72, and 75.9 ± 6.78 out of a maximum of 100, respectively (see Table 1). The scale score range for HSGA ranged from (72.7–100), APT ranged from (53–98), and ACT ranged from (54–98). The mean scores for GPA, HSGA, APT, and ACT across the included undergraduate programs was presented in Table 2. The mean GPA, HSGA, APT and ACT scores the highest was in the occupational therapy program 4.3 ± 0.42, 97.2 ± 2.45, 83.7 ± 4.86 and 77.7 ± 5.67 respectively. However, the lowest score in GPA was in the biomedical instrumentation program (3.7 ± 0.45), and the highest in HSGA was dental technology program (95.7 ± 2.62), nursing program (95.8 ± 3.78), and biomedical instrumentation program (95.9 ± 3.53). The lowest mean score in APT was the nursing program (76.9 ± 6.94), and the lowest mean score in ACT was the nursing program (72.6 ± 7.18).

**Table 2:** description of the study cohort GPA, HSGA, APT and ACT across various programs

Undergraduate Program	GPA Mean ± SD	HSGA Mean ± SD	APT Mean ± SD	ACT Mean ± SD
**Over all programs**	4.1 ± 0.48	97 ± 2.78	81.3 ± 5.72	75.9 ± 6.78
**Minimum value**	2.47	72.7	53	54
**Maximum value**	5.00	100.0	98	98
1. Nursing	3.94 ± 0.46	95.8 ± 3.78	76.9 ± 6.94	72.6 ± 7.18
2. Emergency Medical Services	4.21 ± 0.29	97.2 ± 2.33	82.7 ± 4.57	74.9 ± 5.26
3. Health Education	4.1 ± 0.58	96.8 ± 2.68	80.9 ± 5.39	75.9 ± 6.4
4. Clinical Nutrition	4.2 ± 0.52	97.4 ± 2.36	82.1 ± 5.15	77.1 ± 6.82
5. Clinical laboratory sciences	4.2 ± 0.49	97.5 ± 2.51	82.2 ± 5.75	77.5 ± 6.85
6. Radiological sciences	4.2 ± 0.45	97.4 ± 2.27	81.7 ± 4.75	77.1 ± 5.86
7. Physiotherapy	4.2 ± 0.39	97.4 ± 2.49	82.7 ± 4.49	76.7 ± 6.06
8. Optometry Doctor	4.5 ± 0.26	98.1 ± 1.94	82.6 ± 5.05	76.0 ± 7.43
9. Dental Hygiene	4.1 ± 0.47	97.2 ± 2.96	81.3 ± 4.76	77.4 ± 6.24
10. Dental technology	3.9 ± 0.38	95.7 ± 2.62	78.4 ± 5.8	71.1 ± 6.16
11. Occupational Therapy	4.3 ± 0.42	97.2 ± 2.45	83.7 ± 4.86	77.7 ± 5.67
12. Biomedical Instrumentation	3.7 ± 0.45	95.9 ± 3.53	82.5 ± 4.66	74.7 ± 5.78
13. Speech Therapy	4.2 ± 0.53	97.7 ± 2.15	82.7 ± 5.76	77.7 ± 8.13

Note: *GPA *Grade Point Average at time of graduation, *HSGA *Grade Point Average at Application, *APT *Aptitude Test, *ACT *Achievement Test

Table 3 shows the univariate and multivariate linear regression analysis for the cumulative GPA. The univariate analyses identified gender, type of college, type of program, year of graduation, the HSGA, APT, and ACT tests scores as potential predictors of academic achievement i.e., GPA at graduation (*p* < 0.05). These factors were included in the multivariate linear regression model. The model confirmed that female gender (β = 0.479, 95 % CI = 0.422 to 0.516), CAMS college (β = 0.174, 95 % CI = 0.095 to 0.170), the type of program (β = 0.107, 95 % CI = 0.008 to 0.021), year of graduation; earlier ones (β=-0.087, 95 % CI=-0.077 to -0.030) could predict the Academic achievement. In addition, the higher HSGA (β = 0.237, 95 % CI = 0.035 to 0.049), APT (β = 0.096, 95 % CI = 0.005 to 0.012), and ACT (β = 0.061, 95 % CI = 0.001to 0.008) likely to have a higher cumulative GPA. However, the weigh of variance of years of graduation, APT and ACT are low compared to other factors.

**Table 3:** Univariate and multivariate linear regression analysis for the cumulative GPA

Variable	Univariate linear regression			Multivariate linear regression		
	Linear regression coefficient, β	95 % CI	*p*-Value	Linear regression coefficient, β	95 % CI	*p*-Value
Sex	0.560	0.510–0.589	0.000	0.479	0.422–0.516	0.000
College	-0.081	-0.099--0.025	0.001	0.174	0.095–0.170	0.000
Undergraduate Program	0.080	0.004–0.018	0.001	0.107	0.008–0.021	0.000
Year of Graduation	-0.079	-0.079--0.019	0.001	-0.087	-0.077- -0.030	0.000
HSGA	0.429	0.068–0.083	0.000	0.237	0.035–0.049	0.000
APT	0.243	0.017–0.025	0.000	0.096	0.005–0.012	0.000
ACT	0.387	0.-025-0.031	0.000	0.061	0.001–0.008	0.014

Note: *P*-value significant at *p* < 0.05

Additionally, the R^2^ value was 41.8 %. It indicates that 41.8 % of the variance in GPA scores can predict from the variables gender, type of college, type of program, year of graduation, the HSGA, APT and ACT tests scores.

## Discussion

In this retrospective cohort study, we followed students’ academic achievements and assessed the association between academic achievements using cumulative GPA at time of graduation and other factors including admissions criteria based on HSGA, APT, and ACT among colleges of allied health professions from King Saud University. It has been found that among the quantitative admission methods, the HSGA is the most strongly predictive variable of student achievement, followed by APT and ACT.

Additionally, it is observed that these variables account for 41.8 % of the variation in student GPA. It can therefore be speculated that there is significant variance between the variables and their predictive value, but when combined, they account for a small percentage of the observed variation in GPA.

Previous studies have investigated the predictive value of HSGA, ACT, and APT for the GPA of medical students and found consistent positive correlations [8, 11, 14, 15]. The findings of this study indicate that HSGA is the most predictive of student achievement, and ACT is the weakest predictor. This is inconsistent with previous reports that have investigated the performance of students in medical, dental, and pharmacological colleges. A report assessing the correlation of HSGA, ACT, and APT in medical, dentistry, and pharmacological schools found that ACT is the most predictive parameter for a student’s GPA in the first two years of study [6]. The combination of these three variables was reported to account for 25.5 % of variance in students’ GPA during their first and second years of study. It should be noted that to the best of our knowledge, our study is the first to include students at colleges for allied health professions; further, it featured a large cohort of 1634 individuals, which is higher than most of the studies while also considering cumulative GPA at the time of graduation.

This study showed that students at colleges for allied health professions who scored high on HSGA, ACT, and APT tended to have a higher cumulative GPA. The majority of published studies from Saudi Arabia that have taken up these themes have not considered allied health professions or students’ cumulative GPA at graduation. Student selection at King Saud University relies heavily on cognitive assessments. The findings of the present study indicate that these assessments, which are part of the admissions criteria for King Saud University together with gender, college and program type and year of graduation, accounted for about 41.8 % of variation in GPA. This indicates that several other factors can influence a student’s overall performance. For example, personal qualities play a major role in helping individuals overcome adversity related to these educational programs. Performance self-efficacy has been identified as the strongest correlate for overall student performance [16]. Although a personal interview is a mandatory step in admissions to allied health colleges at King Saud University, it is taken as a means of excluding students who are not physically fit or who do not display a genuine desire to enroll. Thus, the interview results are not weighted in the admission process. Teaching methods and strategies also play an essential role. One study found that parental involvement in education, good and supportive relationships between educators and students, classroom computer technology, and adequate learning facilities are correlated with better academic performance [17]. Autonomous motivation, defined as a genuine interest or personal endorsement, is also associated with better academic achievement [18]. Support-seeking behavior has also been correlated with it [19].

Other lifestyle factors such as sleeping habits also impact the performance of students. These have been shown to have direct and indirect effects, mediated by factors such as conscientiousness, learning or achievement motivation, mood, and alertness [20]. Sleep disturbances are a negative predictor of academic performance [21].

This investigation is subject to a few limitations. First, it uses retrospective observational methods, so causality cannot be inferred. Second, the records included in the analyses were restricted to complete records such as age of the students and were only from King Saud University, so the findings may not be generalizable to other contexts. Another limitation of this study is that overall student performance is reflected by the GPA and is subject to variation between the three colleges, owing to various issues that influence the outcome; therefore, it is important to investigate other factors related to students or the program that might influence students’ academic achievements and its impact on their career in future research. This study assessed the predictors of academic accomplishments for three years cohort. The results of our study could be validated by examining the trends over more years in the future. Additionally, selection criteria could be explored in-depth per program to assist in selecting students who are more likely to perform better academically.

## Conclusions

This study adds to the findings of previous studies that assess correlations among HSGA, ACT, and APT and overall GPA. The results indicate a positive assessment of current admissions criteria, especially at HSGA. These results highlight the need to look into additional strategies that assess noncognitive attitudes and behaviors to improve student selection, thereby improving the quality of healthcare professionals produced. Furthermore, interventional studies are highly encouraged as there is a clear scarcity of these type of research that determine the effectiveness of developed strategies as well as studies that investigate the factors associated with the success of allied health professionals in practice.

## Data Availability

The data set used is locked and stored in the College of Applied Medical Science at King Saud University and can be obtained from the principal investigator on reasonable request.
